# Can the Onset of Type 2 Diabetes Be Delayed by a Group-Based Lifestyle Intervention in Women with Prediabetes following Gestational Diabetes Mellitus (GDM)? Findings from a Randomized Control Mixed Methods Trial

**DOI:** 10.1155/2015/798460

**Published:** 2015-08-18

**Authors:** Angela O'Dea, Marie Tierney, Brian E. McGuire, John Newell, Liam G. Glynn, Irene Gibson, Eoin Noctor, Andrii Danyliv, Susan B. Connolly, Fidelma P. Dunne

**Affiliations:** ^1^School of Medicine, Clinical Sciences Institute, National University of Ireland Galway, Galway, Ireland; ^2^School of Psychology, National University of Ireland Galway, Galway, Ireland; ^3^HRB Clinical Research Facility, National University of Ireland Galway, Galway, Ireland; ^4^Discipline of General Practice, School of Medicine, National University of Ireland Galway, Galway, Ireland; ^5^Croí–The West of Ireland Cardiac Foundation, Croí Heart and Stroke Centre, Moyola Lane, Newcastle, Galway, Ireland; ^6^J.E. Cairnes School of Business & Economics, National University of Ireland Galway, Cairnes Building, Galway, Ireland; ^7^Cardiovascular Medicine, International Centre for Circulatory Health, Imperial College London, London W2 1LA, UK

## Abstract

*Objective*. To evaluate a 12-week group-based lifestyle intervention programme for women with prediabetes following gestational diabetes (GDM). *Design*. A two-group, mixed methods randomized controlled trial in which 50 women with a history of GDM and abnormal glucose tolerance postpartum were randomly assigned to intervention (*n* = 24) or wait control (*n* = 26) and postintervention qualitative interviews with participants. *Main Outcome Measures*. Modifiable biochemical, anthropometric, behavioural, and psychosocial risk factors associated with the development of type 2 diabetes. The primary outcome variable was the change in fasting plasma glucose (FPG) from study entry to one-year follow-up. *Results*. At one-year follow-up, the intervention group showed significant improvements over the wait control group on stress, diet self-efficacy, and quality of life. There was no evidence of an effect of the intervention on measures of biochemistry or anthropometry; the effect on one health behaviour, diet adherence, was close to significance. *Conclusions*. Prevention programmes must tackle the barriers to participation faced by this population; home-based interventions should be investigated. Strategies for promoting long-term health self-management need to be developed and tested.

## 1. Introduction

Women with a history of gestational diabetes (GDM) are at increased risk of developing type 2 diabetes compared to those with normoglycaemic pregnancies [1]. Studies investigating glucose function in the early postnatal period have shown the prevalence of type 2 diabetes to be as high as 38% in the first year postpartum [2] and as high as 60% in women followed for up to 16 years postpartum [3]. The postpartum period is a very important time in determining the future risk of type 2 diabetes in women with GDM; thus there is a strong rationale for preventive interventions at this time.

Some risk factors associated with the development of type 2 diabetes are nonmodifiable such as such as age, ethnicity, or a family history of diabetes. However, some risk factors such as weight, elevated blood glucose, and triglycerides are potentially modifiable though diet, exercise, and lifestyle change [4]; for example, modest weight loss has been shown to be effective in reducing the risk of developing type 2 diabetes in at-risk populations [5].

Large scale randomised controlled trials (RCTs) have shown that the onset of type 2 diabetes can be prevented or delayed by lifestyle intervention in high-risk cohorts [6–8]. However, these studies include mixed gender (usually older) adults. A subgroup analysis of post-GDM women from the Diabetes Prevention Programme showed a 50% reduction in diabetes incidence with lifestyle intervention versus placebo in women at a mean of 12 years following the GDM pregnancy [9]. Evidence to support the efficacy of lifestyle intervention in women with a recenthistory of GDM is however lacking.

A limitation of the above literature is the lack of attention to psychosocial risk factors in lifestyle interventions to prevent diabetes. Factors such as mood, self-efficacy beliefs, and social support have been shown to be associated with prediabetes and type 2 diabetes [10], diabetes related self-care [11], and willingness to engage in lifestyle change [12]. Moreover lifestyle intervention has been associated with positive changes in mood and health outcomes in mixed gender groups with diabetes risk factors [13, 14]. There is a need to investigate the long term sustainability of such effects, particularly in women with previous GDM.

Thus research is required which investigates (i) the efficacy of lifestyle intervention in improving the risk factors associated with the development of type 2 diabetes in women with a recent history of GDM, (ii) the role of psychosocial factors in facilitating behavior change, and (iii) the long term sustainability of behavioural improvements. This study sought to answer these questions through mixed methods RCT to evaluate the effectiveness of an intensive 12-week group based lifestyle intervention programme,* MyAction* [15], as compared with standard care, in reducing diabetes risk factors in women 1–3 years after GDM. The* MyAction* programme uses a combination of education, exercise classes, and cognitive-behavioural approaches to empower individuals and families to make sustainable lifestyle changes. The programme has been shown to be effective in reducing cardiovascular risk factors [16]; but it has not been assessed in women with prior GDM.

In this study, therefore, the effectiveness of the* MyAction* programme was measured in terms of a reduction in the modifiable biochemical, anthropometric, behavioural, and psychosocial risk factors associated with the development of type 2 diabetes in women with prior GDM and persistent glucose abnormality postpartum. Postintervention qualitative interviews with participants will provide context to the findings.

## 2. Materials and Methods

Ethical approval for the study was obtained in March 2012 from the Clinical Research Ethics Committee of Galway University Hospital, part of Ireland's Health Service Executive (HSE).

### 2.1. Participants

Potential participants were identified from the Atlantic Diabetes in Pregnancy (DIP) research database and the pregnancy service of Galway University Hospital Group. Women with a recent history of GDM (i.e., diagnosed GDM in the past 1–3 years) by International Association of Diabetes and Pregnancy Study Group (IADPSG) diagnostic criteria [17] were contacted by letters and follow-up phone calls. The recruitment and randomisation process is described in detail in the trial protocol [18].

Inclusion criteria were at least one of the following at study entry: (1) impaired fasting glucose (IFG) (fasting plasma glucose levels of 5.6−6.9 mmol/L); (2) impaired glucose tolerance (IGT) (two-hour plasma glucose levels of 7.8−11.0 mmol/L); (3) insulin resistance (IR) based on homeostasis model assessment (HOMA2-IR ≥ 1.7) plus at least two of the following risk factors: (a) blood pressure > 130/80 mmHg, (b) total cholesterol > 4.5 mmol/L, (c) LDL cholesterol >2.5 mmol/L, (d) triglycerides >1.69 mmol/L, (e) HDL cholesterol <1.29 mmol/L, (f) obesity (defined as BMI >30 kg/m^2^), and (g) waist circumference >88 cm. Exclusion criteria were (i) type 2 diabetes; (ii) current pregnancy; (iii) insufficient English language fluency to understand the programme content. The recruitment process spanned 10 months, from June 2012 to March 2013.

### 2.2. Description of Intervention/Comparison Group

The study design was a two-group, parallel RCT. Following baseline assessments, eligible participants were randomly assigned to the intervention or wait control group in an equal ratio of 1 : 1. An independent researcher was responsible for generating the allocation sequence and for assigning participants to the intervention groups. Those assigned to the intervention group received the 12-week intensive lifestyle programme,* MyAction*. Full background to the* MyAction* programme is provided in the trial protocol [18]. Briefly, the programme is delivered by a multidisciplinary team of nurses, dieticians, and physical activity specialists and is supported by a physician. The programme includes an initial individualised assessment followed by 12 weekly sessions of 2.5 hours each week comprising of a one-hour group exercise programme, a group education seminar, and a one-to-one session involving a motivational interview and individual goal setting with a specialist nurse, physiotherapist, or dietician. The wait control group receives the standard health care advice provided to women with previous GDM. In this study, standard care is defined as (i) educational pamphlets for reducing diabetes risks and (ii) routine follow-up by the participant's own general practitioner.

### 2.3. Outcomes

The primary outcome measure is the mean change in FPG levels from the time of the baseline assessment to the one-year follow-up assessment. Secondary outcomes were mean change in 2-hour postload glucose tolerance (GT2h), insulin resistance (IR), diet adherence, weight and waist circumference, physical activity, fitness and lipid profile and measures of mood, cognition, and wellbeing. Participants in the intervention cohort also underwent evaluation for some secondary outcomes immediately following the intervention.  Table 1 outlines the definitions, measurement techniques, and time points for each of the outcomes.

### 2.4. Sample Size and Power Calculation

The sample size required for the RCT was 54 participants. This number was calculated based on estimates provided from pilot data from women with a history of GDM (*n* = 74) receiving conventional health care. The pilot group had a standard deviation of 0.64 mmol/L for the difference in FPG on an OGTT between two time points: approximately three months postpregnancy and then one to three years later. Given the standard deviation, it was estimated that a sample size of 27 in each study arm was necessary to have 80% power (at the 0.05 significance level) to detect a mean difference of 0.5 mmol/L in FPG between baseline and one-year follow-up in the two study arms.

### 2.5. Statistical Analysis

Suitable numerical (mean and standard deviation) and graphical summaries (box and scatterplots) were used to compare the groups at baseline and to provide interval estimates of the mean difference at end of programme assessment (EOP) (on a subset of variables) and at one-year follow-up on all measured variables. A linear model (ANCOVA) was used to investigate the effect of intervention on the change in glucose function at follow-up, while adjusting for baseline as a covariate and for patient characteristics; subsequently, ridge regression was used to adjust for multicollinearity between covariates. All analyses were performed as intention to treat analysis. All analyses were carried out using the software packages R (version 3.0) and Minitab 16 Statistical Software. All model assumptions were assessed using suitable residual plots.

### 2.6. Qualitative Methods and Analysis

Semistructured interviews were conducted with 17 trial participants who were randomized to the intervention group. Of those, 12 completed the intervention and 5 were noncompleters. An interview guide with open ended questions was used to elicit respondents experiences of the intervention, barriers, and facilitators to lifestyle change, social support, efficacy beliefs, and beliefs about optimal interventions for this population. All of the interviews were conducted face-to-face at the time of the one-year follow-up assessment. All interviews were digitally recorded with the permission of each participant and were transcribed verbatim. Interview transcripts were analysed thematically using an inductive approach [19]. The transcripts were read and reread and noteworthy aspects of the data were systematically coded. Then the coded text was organised into broad themes. Following this, the themes were reviewed, refined, and named.

## 3. Results

Out of 410 women who received information about the trial, 89 agreed to participate and were assessed for eligibility. Of these, 50 met eligibility criteria for inclusion into the trial. Of the 50 eligible participants, 26 were randomised to the* MyAction* lifestyle intervention programme and 24 to the wait control group, who received standard care.

### 3.1. Attendance Rates

**Figure 1:**
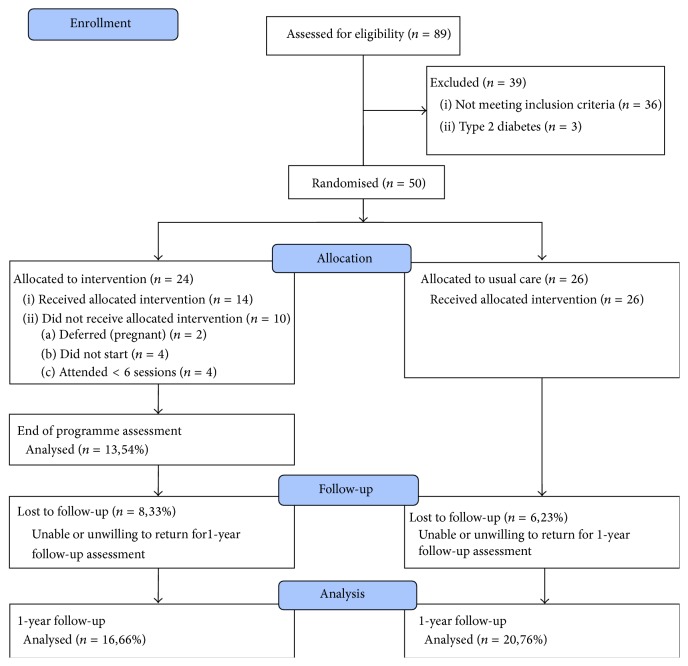
Consort flow diagram showing flow of participants through the trial.

Of the 24 participants randomized to the intervention group, 14 (58%) were deemed to have completed the intervention with attendance rates ≥6 sessions. The average number of sessions attended by the completers was 9.5. The remaining 10 participants (42%) either did not start the intervention (*n* = 4), had attendance rates <6 sessions (*n* = 4), or deferred due to pregnancy (*n* = 2). Of the intervention completers (*n* = 14), 13 attended EOP assessment which took place immediately following completion of the programme, and all 14 completers attended the one-year follow-up assessment which took place one year following programme commencement. Two noncompleters also attended one-year follow-up assessment and are included in the ITT analysis. Loss to follow-up was 33% for the intervention group and 23% for the control group. The flow diagram in Figure 1 represents the movement of participants through the stages of the study.

### 3.2. Baseline Analysis

**Figure 2:**
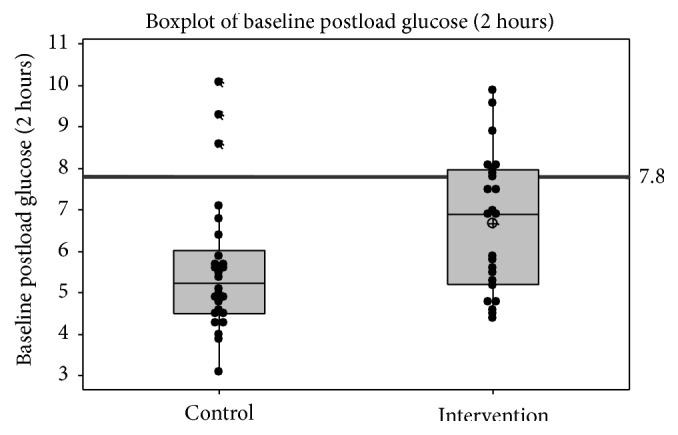
Boxplot of baseline glucose tolerance (2-hour) (GT2h) per group.

A comparison of control and intervention groups at baseline (see Table 2) revealed that groups were comparable on all measured variables except GT2h, which was significantly higher for the control than for the intervention group (*p* = 0.025). The boxplot in Figure 2 shows individual values on GT2h in both groups. Thus randomisation was not successful in ensuring that groups were comparable on baseline glucose tolerance. Participants in both groups are within the normal ranges on lipid profile; however their weight is high and, resultantly, BMI and waist circumference are well above the recommended range. Exercise per week is below the recommended range of 150 minutes of moderate intensity exercise per week. Participants in both groups scored approximately 6 out of a possible 14 for baseline adherence to the recommended diet. In terms of fitness participants in both groups have a METmax score of approximately 8; this is slightly below the desirable level of 9 which is associated with lowest risk [20]. On the psychosocial variables, participants in this study scored less favorably than population norms on energy and vitality (EVI) and on psychological distress [21], but within the normal range on depression anxiety and stress [22]. On general health and quality of life participants scored in the middle of the range, indicating median levels of satisfaction on these variables [23]. Mean levels of social support from family, friends, and a significant other were comparable to previously reported studies [24, 25]. In terms of motivation to change, respondents score in the middle range on a 5-point scale which represents an intention to change but no initiation of change behaviour. Scores on exercise and diet self-efficacy fall in the middle range and are comparable to those reported elsewhere [13].

### 3.3. Analysis of Improvement from Baseline to End of Programme (EOP)

The intervention group was tested at EOP on a subset of variables. Analysis of improvement from baseline to EOP revealed significant improvement in weight, BMI, waist circumference, fitness (METmax), total cholesterol, and LDL cholesterol. There was a significant disimprovement in mean HDL cholesterol (*p* = 0.02) and no significant change in mean triglycerides (*p* = 0.54).  Table 3 shows improvement from baseline to EOP for (*n* = 13) participants who completed the intervention and returned for EOP follow-up testing. In each case, positive scores represent improvement, and negative scores represent disimprovement.

### 3.4. Analysis of Improvement from Baseline to One-Year Follow-Up

#### 3.4.1. Biochemistry

Paired *t*-tests using nonadjusted scores revealed no evidence of a significant intervention effect on the primary outcome, FPG values (*p* = 0.36), or on IR (*p* = 0.94); however, there was evidence of a significant improvement in the intervention group on GT2h (*p* = 0.02). There were no significant differences between groups on lipid profile. Linear regression models (ANCOVA), adjusting for baseline and other participant characteristics (BMI, cholesterol, and fitness), were used to estimate the effect of the intervention on FPG, GT2h and IR. A significant intervention effect was found for GT2h (*p* = 0.03), but not for FPG (*p* = 0.67) or insulin resistance (*p* = 0.33). However, when a ridge regression penalty was introduced in order to adjust for multicollinearity between covariates, no significant effect of the intervention was found on FPG (*p* = 0.44), GT2h (*p* = 0.16), or IR (*p* = 0.27). There was, however, an effect of fitness at baseline on GT2h with higher levels of fitness (METmax) being associated with greater improvement (*p* = 0.01).

#### 3.4.2. Anthropometry

There were no significant differences in improvement between groups on weight, BMI, or waist circumference. An ANCOVA model revealed no significant intervention effect on BMI after adjusting for baseline BMI and other participant characteristics: mood, cognition, wellbeing, diet adherence, and fitness levels (*p* = 0.57). When ridge regression penalty was introduced in order to adjust for multicollinearity between covariates, no significant effect of the intervention was found for BMI (*p* = 0.91); however there was a significant negative effect of depression at baseline on improvement in BMI (*p* = 0.05), with higher depression being associated with lower levels of improvement.

#### 3.4.3. Behaviour

There were no significant differences between groups on diet adherence or physical activity. ANCOVA models revealed no significant intervention effect on diet adherence (*p* = 0.53) or physical activity levels (*p* = 0.33) after adjusting for baseline mood, cognition, and wellbeing. However, ridge regression analysis revealed that the effect of the intervention on diet adherence was nearing significance (*p* = 0.07); the effect of family support on this outcome was also nearing significance (*p* = 0.06). There was no significant effect of intervention on physical activity levels (*p* = 0.38), nor was there an effect of baseline mood, cognition, or wellbeing on this outcome.

#### 3.4.4. Psychosocial

On the psychosocial variables, significant differences between groups were observed on stress, diet self-efficacy, and quality of life with the intervention group improving significantly over the control group.  Table 4 displays the *p* values and confidence intervals for analysis of differences. In each case, positive scores represent improvement and negative scores represent disimprovement.

### 3.5. Qualitative Results

Below the attitudes and opinions of intervention group “completers” and “noncompleters” are presented.

#### 3.5.1. Reasons for Dropping out and Nonattendance

*Child Care Responsibilities. *For the women who did not complete the intervention and for several women who did complete it, childcare responsibilities were cited as the main barrier to attendance. Some women had no one to leave their children to, and others did not want to leave their children. Approximately 50% of the women in the study worked outside of the home; many of them expressed feeling guilty about leaving the children for a full evening each week.

*It's just not an option for me; I do not have anyone to leave the children to.*

*I feel I need to be at home to do the homework with the children and put them to bed.*

* I could not come home from a long shift at work and pick her up from her childcare and go out again straight away.*

*Time and Travel*. For the women who did not complete the intervention, lack of time was cited as the main reason for dropping out. In addition several women stated that they could not commit to a regularly scheduled time or that the program took too much time. For women from rural locations, the travel time was also a barrier to attendance.

*The main reason I gave up coming was time, it took up my whole night.*

*Could not make the regularly scheduled times.*

*I tried to fit it into my schedule but the drive was really too far.*

*Not Prioritising Oneself*. Many of the participants expressed difficulties in prioritizing themselves and their health over the needs of their families. In many cases the educational, social, and exercise needs of their children and their partners took precedence over their own health needs.

*You cannot both leave the house.*

*I feed my children healthy food but I do not take care of myself.*

#### 3.5.2. Facilitators to Attendance

*Support from Partner.* Support from a significant other, specifically the life partner or husband, was critically important for the women in this study. None of the women who did not have a partner were able to complete the program. For those who completed, the support of their partner was crucial to their ability to take time away from the family. The women in this study preferred not to ask extended family members to support in childcare in order for them to participate in the intervention.

*I couldn't have done it if my husband hadn't been supportive of it.*

*My children are my responsibility, I do not ask other people to mind them unless it's an emergency.*

#### 3.5.3. Motivation to Change

*Weight Loss. *For all of the women, the main motivation cited for participation in the program was to lose weight. Improving diabetes risk factors and general health concerns were a secondary motivation.

*My problem always needs to get solved; I always need to lose weight.*

*Accountability. *The weekly one-to-one interview and the “weigh-in” emerged as the key motivators for participants. Accountability to the health care team was a strong motivator to adhere to health goals.

*I knew that I would be getting weighed-in each week and having to be accountable to the staff.*

*The program was the motivation.*

#### 3.5.4. Programme Benefits

*Stress, Mood, and Wellbeing.* Participants that completed the intervention reported improvements in their mood and self-confidence as a result of the program. Improved confidence is particularly reported in two areas (i) the confidence to exercise vigorously and (ii) the confidence to prioritise their own needs along with the needs of their family. It seems that taking the time to attend the program empowered the women to take time away from the family for their social and health needs. For many respondents this is something they had not done since the birth of their children.

*It helped me to be more positive about taking care of myself and it gave me some positive techniques about fitting in exercise.*

*It gave me the confidence to leave for the whole evening.*

*It has kept my spirits up; from a morale point of view I won't underestimate it.*

*It really has helped me get over a lot of stresses.*

*Exercise Habit. *For some women who completed the program the benefits of the program appear to be sustained beyond the duration of the intervention. For these women regular exercise has become a daily habit, and they are feeling the benefits both physically and psychologically. A number of the women started running on a regular basis, something many of them had previously not considered as an option.

*I am getting out a lot more, I was doing nothing beforehand.*

*The biggest benefit I got from the program is the confidence that I can run. *

*It spurred me on to running; I find that I love running.*

#### 3.5.5. Limitations of the Programme

*Lack of Ownership or Self-Management. *For some participants, even those that were successful in losing weight throughout the duration of the study, maintaining improvements to diet and exercise after the programme ended was a challenge. Many women could not sustain the lifestyle changes without the support of the intervention.

*When it finished I just went back to square one really.*

*Without someone to check on you and coax you and motivate you, the motivation goes.*

*I found it hard to keep it up after the program.*

* I stopped exercising once program stopped.*

*I did well, I lost a good bit of weight but I have it all on again.*

#### 3.5.6. Optimal Lifestyle Intervention

When asked their opinions about and “ideal” lifestyle intervention program all of the women stated that the program must be accessible and flexible. Many women stated that online or web based programmes would work well for them as they could engage in the program at times that suited them. However they also valued one-on-one consultations with healthcare professionals in order to engage in joint goal setting and performance monitoring.

*I think an online program would work for me.*

*Something that is flexible, that I could do whenever I liked.*

## 4. Discussion

This study is the first to assess the efficacy of a group-based intervention specifically in women with a recent history of GDM. Before discussing the findings of the trial two important limitations should be noted. First, this trial included relatively low number of participants due to recruitment and retention challenges with this population [26]. Although we almost reached our sample target of 54 participants, 42% of those randomized to the intervention group did not complete the intervention. Thus, assessing the true efficacy of this intervention is impeded. Second, randomisation was not successful in ensuring that groups were equivalent in terms of glucose dysfunction at baseline; here the intervention group had higher mean GT2h than controls; adjustments were made for this difference in the multivariate analysis.

The findings of the trial reveal that the intervention had the greatest impact on psychosocial factors. At the time of the 1-year follow-up, the intervention group showed significant improvements over the control group on stress, diet self-efficacy, and quality of life. There was no evidence of a long term effect of the intervention on measures of biochemistry or anthropometry; however, there was evidence of a potential effect of the intervention on diet adherence at one-year follow-up. The intervention group did show significant improvements on weight, waist circumference, diet adherence, fitness, and cholesterol at EOP; however these improvements were not sustained at one-year follow-up, suggesting that the women did not develop the skills to self-manage their health in the postintervention period. Baseline fitness levels, lower depression, and family support were associated with health improvements.

The findings of the qualitative analysis concur with the quantitative trial outcomes. While the impact of the intervention on objective health measures is equivocal, for many women who completed the intervention, it had a meaningful and positive impact on their psychosocial health. Simply making the decision to partake in the intervention was empowering to the women as this represented a decision to prioritise their own health needs alongside the needs of their families and provided them with a license to take time away from the family for their own social and health needs. Support from a partner was critical to the women's ability to take time away from the family to partake in health enhancing activity outside of the home. For others though, particularly those with less social support, group based interventions are not the panacea for their long term health management needs and many could not sustain changes in lifestyle after the intervention ended; for these women the long term benefits were minimal.

These findings are supported in the literature, for example, other evaluations of behavioural intervention to enhance weight loss in postpartum women [27]; Kim et al. 2012 [28, 29] also report limited or no significant differences in postpartum weight loss, diet adherence, or physical activity as a result of lifestyle intervention. In each case, low levels of participation are identified as an important limiting factor. Studies investigating barriers and facilitators to participation in lifestyle change also identify a lack of assistance with childcare and insufficient time as the most common barriers to physical activity in postpartum women. Facilitators include high social support and high self-efficacy and access to childcare [30–32].

Thus, women in the early postpartum period face multiple barriers to participation in group based lifestyle intervention. Optimal approaches for preventative measures for this population must first and foremost tackle the issue of barriers to attendance and participation faced by this population. Evidence from this and other trials suggests that home-based interventions via mail, telephone, or internet/email may be more feasible and successful in this population.

In addition to the delivery mechanism, program content must also be appropriate for the population; the evidence from this study highlights two principles upon which future interventions should be based: (i) Women with persistent glucose dysfunction following GDM have an inadequate understanding of their health risks, and education on the health risks associated with prediabetes should be included in any intervention with this population. (ii) Strategies that promote self-directed behaviour change must be incorporated into programme design. The challenge facing researchers will be to develop internet based intervention programmes that fulfill these requirements; such programs should be developed and tested.

## 5. Conclusions

For some women group or community based lifestyle intervention programmes can have life affirming effects and lead to positive outcomes. For others though, particularly those with less social support, group based interventions are not the panacea for their long term health management needs. It is pertinent now to investigate whether home based interventions that are administered via mail, telephone, or internet/email may be more feasible and successful in this population. Such programmes should be based on an understanding of the role of psychosocial factors in facilitating or ameliorating the effectiveness of lifestyle intervention, and programmes should be tailored to population and aim to develop the skills for health self-management in this population.

## Figures and Tables

**Table 1:** Primary and secondary outcome measures and measurement time points.

Outcome	Definition and measurement	Timepoint measured		
		Baseline	EOP	1-year
*Primary outcome *				
Fasting plasma glucose (FPG 0-hour)	Reduction in FPG (0-hour) on a 75 gram oral glucose tolerance test (OGTT)	✓		✓
*Secondary outcomes *				
Glucose tolerance (GT2 hour)	Reduction in postload glucose tolerance (2-hour) on a 75 gram OGTT	✓		✓
Insulin resistance (IR)	Reduction in IR as measured by Homeostasis Model Assessment (HOMA2- IR), using fasting glucose and insulin values [33] on a 75 gram OGTT	✓		✓
Lipid profile	(i) Reduction in triglycerides, LDL, and total cholesterol (ii) Improvement in HDL cholesterol	✓	✓	✓
Diet adherence	Improvement in overall Mediterranean Diet Score, a composite diet index based on the traditional Mediterranean dietary pattern [34]	✓	✓	✓
Weight and shape	(i) Reduction in weight (kilograms) (ii) Reduction in waist circumference (CM)	✓	✓	✓
Physical activity and fitness	(i) Total exercise per week (frequency × duration) (ii) Change in cardiorespiratory fitness measured using maximal metabolic equivalent (METmax) on an objective physical fitness test (Chester Step Test) [35]	✓	✓	✓
Mood	(i) Positive mental health: 4 items from RAND SF-36 questionnaire addressing affective aspects of wellbeing [21] (ii) Nonspecific psychological distress: 5 items from the Mental Health Index-5 (MHI-5) in the RAND SF-36 questionnaire [21] (iii) Depression, anxiety, and stress: 21 items from Depression, Anxiety, and Stress Scale (DASS-21) [22]	✓		✓
Cognition	(i) Perceived social support: Multidimensional Scale of Perceived Social Support from family, friends, and a significant other [25] (ii) Motivation to change: a one item forced choice question with 5 options to assess current stage of change [13] (iii) Diabetes-related self-efficacy: 18-item assessment of confidence to engage in exercise and healthy eating under different “barrier” conditions [13]	✓		✓
Wellbeing	(i) General health: stand-alone measure of self-rated health included in the Irish Survey of Lifestyle, Attitudes, and Nutrition (SLÁN), 2007 [23] (ii) Quality of life: single-item assessment included in SLÁN 2007 [23]	✓		✓

**Table 2:** Baseline values on all measured variables.

Variable	Control (*n* = 26)	Intervention (*n*= 24)	*p* value	Confidence interval
	Baseline mean (sd)	Baseline mean (sd)		
Glucose function				
Fasting plasma glucose	5.36 (0.59)	5.37 (0.54)	0.95	−0.33, 0.31
Glucose tolerance (2 hours)^†^	5.60 (1.65)	6.69 (1.66)	0.02	−2.03, −0.14
Insulin resistance (HOMA)	3.02 (1.19)	2.62 (1.19)	0.25	−0.30, 1.09
Lipids profile				
Triglycerides	1.59 (0.69)	1.26 (0.65)	0.08	−0.05, 0.71
HDL cholesterol	1.34 (0.37)	1.39 (0.35)	0.60	−0.26, 0.15
LDL cholesterol	3.17 (0.89)	2.96 (0.79)	0.38	−0.27, 0.69
Total cholesterol	5.24 (1.03)	4.95 (0.76)	0.26	−0.22, 0.80
Weight and shape				
Weight	97.99 (19.91)	93.25 (16.62)	0.36	−5.66, 15.15
BMI	35.53 (6.86)	35.49 (6.25)	0.98	−3.70, 3.77
Waist circumference	115.42 (19.12)	112.0 (14.07)	0.48	−6.61, 12.6
Physical activity and diet				
Total PA per week	82.7 (103.1)	66.7 (96.9)	0.54	40.9, 72.9
Estimated METmax	8.59 (1.75)	7.97 (1.57)	0.19	−0.33, 1.57
Mediterranean diet score	6.08 (2.33)	6.62 (2.34)	0.41	−1.87, 0.78
Mood				
Energy and vitality (EVI)	44.81 (22.34)	48.54 (19.42)	0.53	−15.68, 8.21
Negative PD (NPD)	67.23 (16.16)	66.00 (17.97)	0.80	−8.47, 10.93
Depression	5.31 (5.48)	4.67 (4.13)	0.72	−2.29, 3.26
Anxiety	3.81 (4.06)	3.67 (3.42)	0.83	−1.93, 2.36
Stress	6.96 (5.18)	6.63 (4.92)	0.85	−2.59, 3.11
Cognition				
Motivation to change (MTC)	3.53 (0.86)	3.25 (0.94)	0.26	−0.22, 0.80
Social support: significant other	6.43 (2.88)	5.72 (7.18)	0.06	−0.21, 5.92
Social support: family	5.28 (7.59)	5.12 (6.06)	0.75	−3.31, 4.54
Social support: friends	5.43 (4.42)	5.12 (6.16)	0.41	−1.80, 4.30
Exercise self-efficacy	3.39 (0.93)	3.14 (0.89)	0.21	−0.19, 0.83
Diet self-efficacy	3.38 (0.85)	3.01 (0.77)	0.19	−0.16, 0.78
Wellbeing				
General health (GH)	2.92 (0.89)	2.95 (0.95)	0.38	−0.23, 0.59
Quality of life (QOL)	3.81 (0.56)	3.62 (0.87)	0.89	−0.56, 0.48

^†^ *p* < 0.05.

**Table 3:** Improvement from baseline to EOP (intervention group only).

Variables assessed at EOP	Baseline mean (sd)	EOP mean (sd)	Improvement (sd)	*p* value	95% confidence interval for mean improvement
Weight (kg)^‡^	90.71 (13.30)	88.09 (13.98)	2.62 (2.87)	0.006	(0.88, 4.36)
BMI^†^	34.98 (4.52)	33.93 (4.57)	1.04 (1.55)	0.03	(0.10, 1.98)
Waist circumference (cm)^†^	112.3 (14.69)	107.63 (18.13)	4.68 (5.11)	0.016	(1.25, 8.11)
Med diet score^*∗*^	6.92 (2.29)	7.84 (3.21)	0.92 (2.53)	0.21	(−2.45, 0.60)
Estimated METmax^*∗*‡^	8.17 (1.36)	9.59 (1.58)	1.42 (0.87)	0.001	(0.86, 1.97)
Total cholesterol^†^	5.08 (0.68)	4.40 (0.46)	0.68 (0.80)	0.013	(0.13, 1.22)
Triglycerides	1.23 (0.59)	1.32 (0.68)	−0.09 (0.48)	0.54	(−0.41, 0.23)
HDL cholesterol^*∗*†^	1.41 (0.35)	1.21 (0.28)	−0.20 (0.25)	0.02	(−0.36, −0.03)
LDL cholesterol^†^	3.07 (0.70)	2.60 (0.43)	0.47 (0.60)	0.03	(0.03, 0.90)

^†^ *p* < 0.05, ^‡^ *p* < 0.01.

^*∗*^On these variables higher scores at EOP represent an improvement in function. Thus improvement is calculated by subtracting baseline score from EOP score. On all other variables a lower score at EOP represents improvement; therefore, improvement is calculated by subtracting EOP score from baseline score.

**Table 4:** Improvement from baseline to one-year follow-up (by group).

Variable	Improvement control (*n* = 20) mean (sd)	Improvement intervention (*n* = 16) mean (sd)	*p* value	95% CI for difference in mean improvement
Glucose function				
Fasting plasma glucose (0 h)	−0.13 (0.63)	0.04 (0.45)	0.36	(−0.54, 0.20)
Glucose tolerance (2 h)^†^	−0.27 (1.46)	0.81 (1.21)	0.02	(−1.99, −0.18)
Insulin resistance	0.41 (1.21)	0.45 (1.63)	0.94	(−1.15, 1.07)
Lipids profile				
Triglycerides	0.09 (0.47)	−0.14 (0.76)	0.31	(−0.23, 0.68)
HDL cholesterol^*∗*^	0.02 (0.28)	−0.11 (0.20)	0.13	(−0.04, 0.29)
LDL cholesterol	0.33 (0.64)	0.34 (0.59)	0.96	(−0.43, 0.41)
Total cholesterol	0.23 (0.44)	0.39 (0.84)	0.50	(−0.65, 0.33)
Weight and waist circumference				
Weight (kg)	0.08 (5.66)	0.84 (4.93)	0.67	(−4.36, 2.83)
BMI	−0.10 (2.19)	0.19 (1.83)	0.67	(−1.65, 1.08)
Waist circumference (cm)	−0.01 (9.18)	0.81 (7.24)	0.77	(−6.48, 4.83)
Physical activity and diet				
Total physical activity per week (mins)^*∗*^	52.0 (103)	−9.2 (93)	0.10	(−12.6, 134.6)
Estimated METmax^*∗*^	0.13 (1.46)	0.99 (1.68)	0.13	(−0.27, 1.99)
Mediterranean diet score^*∗*^	0.62 (2.22)	0.00 (2.06)	0.39	(−.82, 2.06)
Mood				
Energy and vitality	−3.36 (18.25)	1.56 (5.21)	0.37	(−15.68, 8.21)
Nonspecific psychological distress	3.24 (15.37)	8.00 (15.93)	0.51	(−8.47, 10.93)
Depression	1.90 (3.42)	3.43 (3.81)	0.21	(−2.29, 3.27)
Anxiety	0.90 (2.88)	1.31 (2.38)	0.64	(−1.93, 2.37)
Stress^†^	0.95 (2.11)	3.31 (3.94)	0.04	(−4.62, −0.11)
Cognition				
Motivation to change^*∗*^	0.33 (1.19)	0.75 (1.43)	0.35	(−0.22, 0.80)
Social support: significant other^*∗*^	0.09 (3.98)	2.50 (7.00)	0.23	(−0.21, 5.92)
Social support: family^*∗*^	0.43 (4.26)	1.62 (3.61)	0.27	(−3.31, 4.54)
Social support: friends^*∗*^	0.38 (4.18)	2.07 (4.54)	0.38	(−1.80, 4.31)
Exercise self-efficacy^*∗*^	−0.21 (1.19)	0.02 (0.91)	0.33	(−0.19, 0.83)
Diet self-efficacy^*∗*†^	−0.52 (1.06)	0.08 (0.72)	0.04	(−1.19, −0.008)
Wellbeing				
General health^*∗*^	−0.04 (0.59)	0.31 (0.94)	0.19	(−0.56, 0.49)
Quality of life^*∗*†^	−0.09 (0.83)	0.46 (0.64)	0.02	(−1.061, −0.063)

^†^ *p* < 0.05.

^*∗*^On these variables higher scores at 1-year follow-up represent an improvement in function. Thus, improvement is calculated by subtracting baseline score from 1-year follow-up score. On all other variables a lower score at 1-year follow-up represents improvement; therefore, improvement is calculated by subtracting 1-year follow-up score from baseline score.
